# Deletion of aquaporin-4 increases extracellular K^+^ concentration during synaptic stimulation in mouse hippocampus

**DOI:** 10.1007/s00429-014-0767-z

**Published:** 2014-04-18

**Authors:** Nadia Nabil Haj-Yasein, Cecilie Elisabeth Bugge, Vidar Jensen, Ivar Østby, Ole Petter Ottersen, Øivind Hvalby, Erlend Arnulf Nagelhus

**Affiliations:** 1 Letten Centre, Institute of Basic Medical Sciences, University of Oslo, 0317 Oslo, Norway; 2 Department of Physiology, Institute of Basic Medical Sciences, University of Oslo, 0317 Oslo, Norway; 3 Centre for Molecular Medicine Norway, Nordic EMBL Partnership, University of Oslo, 0318 Oslo, Norway; 4 Centre for Integrative Genetics, Norwegian University of Life Sciences, 1432 As, Norway; 5 Department of Anatomy, Institute of Basic Medical Sciences, University of Oslo, 0317 Oslo, Norway; 6 Department of Neurology, Oslo University Hospital, 0027 Oslo, Norway

**Keywords:** Astrocytes, AQP4, Extracellular space, Glia, K^+^ homeostasis, Synaptic activation

## Abstract

The coupling between the water channel aquaporin-4 (AQP4) and K^+^ transport has attracted much interest. In this study, we assessed the effect of *Aqp4* deletion on activity-induced [K^+^]_o_ changes in acute slices from hippocampus and corpus callosum of adult mice. We show that *Aqp4* deletion has a layer-specific effect on [K^+^]_o_ that precisely mirrors the known effect on extracellular volume dynamics. In CA1, the peak [K^+^]_o_ in stratum radiatum during 20 Hz stimulation of Schaffer collateral/commissural fibers was significantly higher in *Aqp4* ^−*/*−^ mice than in wild types, whereas no differences were observed throughout the [K^+^]_o_ recovery phase. In stratum pyramidale and corpus callosum, neither peak [K^+^]_o_ nor post-stimulus [K^+^]_o_ recovery was affected by *Aqp4* deletion. Our data suggest that AQP4 modulates [K^+^]_o_ during synaptic stimulation through its effect on extracellular space volume.

## Introduction

Synaptic activity causes release of K^+^ into the extracellular space. The excess K^+^ must be rapidly removed in order to avoid hyperexcitation and epileptic seizures. The mechanisms underpinning K^+^ homeostasis in brain are therefore of critical importance for brain function.

A number of membrane transporters and channels contribute to clearance of K^+^ from the extracellular space. Foremost among these are the Na,K-ATPase, which is responsible for a major fraction of the energy expenditure in brain, and the Kir4.1 inwardly rectifying K^+^ channel (Kofuji and Newman 2004). With the discovery of the brain water channel aquaporin-4 (AQP4), the question arose whether this aquaporin could be involved in K^+^ clearance (Nielsen et al. 1997). This hypothesis was strengthened by the finding that AQP4 was colocalized with Kir4.1 in endfeet of retinal Müller cells (Nagelhus et al. 1999) and that selective removal of AQP4 from endfoot membranes delayed K^+^ clearance following high-frequency activation of hippocampal synapses (Amiry-Moghaddam et al. 2003). Delayed K^+^ clearance was similarly observed following *Aqp4* deletion, albeit only when [K^+^]_o_ was mildly increased (Strohschein et al. 2011).

It has long been known that any effect of AQP4 on K^+^ clearance must be indirect, as AQP4 is impermeable to K^+^ and other ions (Nagelhus and Ottersen 2013). Also, removal of AQP4 fails to affect the Kir4.1 conductance under basal conditions (Zhang and Verkman 2008). Some authors have pointed to a possible interaction between AQP4 and the Na,K-ATPase (Illarionova et al. 2010; Strohschein et al. 2011). Adding to the complexity, Strohschein et al. (2011) showed that *Aqp4* deletion enhances gap-junctional coupling, which would facilitate K^+^ redistribution through the astroglial syncytium.

While several studies have explored the effect of *Aqp4* deletion or AQP4 mislocalization on K^+^ clearance (Amiry-Moghaddam et al. 2003; Padmawar et al. 2005; Binder et al. 2006; Strohschein et al. 2011; Thrane et al. 2013), it remains to resolve whether AQP4 regulates [K^+^]_o_ at the synaptic level during afferent stimulation. The importance of this question derives from the finding that *Aqp4* deletion is associated with an increased severity of epileptic seizures (Binder et al. 2006). Here, we show that *Aqp4* ^−/−^ animals exhibit a more pronounced [K^+^]_o_ peak than wild types during 20 Hz stimulation of Schaffer collateral/commissural fibers. We argue that the increased [K^+^]_o_ peak reflects altered volume dynamics during synaptic stimulation.

## Materials and methods

### Animals

Studies were conducted with adult (8–18 weeks, weighing 20–30 g) constitutive *Aqp4* ^−/−^ mice (Thrane et al. 2011) and wild types of both sexes. The experiments comply with Norwegian laws and were approved by the Animal Care and Use Committee of Institute of Basic Medical Sciences, University of Oslo.

### Electrophysiology

#### Slice preparations

Wild type and *Aqp4* ^−/−^ mice were euthanized with Suprane (Baxter) and brains were removed. Transverse slices (400 μm) from the dorsal and middle portion of each hippocampus, or coronal slices of the cerebrum containing corpus callosum (400 μm), were cut with a vibroslicer in artificial cerebrospinal fluid (ACSF, 4 °C, bubbled with 95 % O_2_ and 5 % CO_2_, containing (in mM): 124 NaCl, 2 KCl, 1.25 KH_2_PO_4_, 2 MgSO_4_, 2 CaCl_2_, 26 NaHCO_3_, and 12 glucose. Both in the resting and interface recording chambers, slices were continuously exposed to humidified gas at 28–32 °C and perfused with ACSF (pH 7.3).

In some of the hippocampal experiments, we applied 50 μM DL-2-amino-5-phosphonopentanoic acid (AP5; Sigma-Aldrich) and 20 μM 6,7-dinitroquinoxaline-2,3-dione (DNQX; Tocris) to block ionotropic glutamate receptors.

#### Stimulation and recording

Before the experiments, ion-sensitive electrodes were silanized and filled with 150 mM tetramethylammonium chloride (TMA^+^, Sigma Life Sciences). The tips were filled with a liquid K^+^ ion exchanger (IE190; World Precision Instruments) by gentle suction. The electrodes were calibrated by standard solutions of [K^+^] (3, 25, 6, 9, and 12 mM). The log-linear fit was used to calculate the [K^+^]_o_ from each experiment.

In the hippocampus, orthodromic synaptic stimuli (50 μs, <300 μA, 0.1 Hz) were delivered through a tungsten electrode situated in stratum radiatum of the CA1 region. The extracellular synaptic responses were monitored by a reference glass electrode (filled with ACSF) placed close to the ion-sensitive electrode in stratum radiatum or stratum pyramidale at a fixed distance (400 µm) from the stimulation electrode (Fig. 2a, inset). The reference electrode was coupled to the ion-sensitive microelectrode (custom-built differential amplifier, 2 Hz low-pass filter). Thus, the monitored changes in direct current (DC) level reflected the changes in [K^+^]_o_.

Following the presence of stable synaptic responses for at least 10 min, we activated the afferent fibers at 20 Hz for 10 s. A similar design was used when eliciting and recording the extracellular prevolley in the corpus callosum. These electrodes were placed on each side of the sagittal line separated at a constant distance (500 µm) (Fig. 2d, inset).

#### Analysis

A single exponential function (Origin 8) was in each experiment fitted to the [K^+^]_o_ decay following the 20 Hz stimulation train. The decay constant was obtained from each experiment.

Data were pooled across mice of the same genotype and are presented as mean ± standard error of the mean (SEM), unless otherwise indicated. For comparison between genotypes, we used a linear mixed model statistical analysis (SAS 9.2), with *p* < 0.05 being designated as statistically significant.

### Fixation and immunocytochemistry

After recording, the slices were immersion fixed in 0.1 M phosphate buffer (PB; pH 7.4) containing 4 % formaldehyde (4 °C, over night). The slices were then cryoprotected in sucrose (10, 20, and 30 % in PB) and cut in 15-μm sections on a cryostat. Immunocytochemistry was carried out using an indirect fluorescence method (Nagelhus et al. 1999). The concentrations of the antibodies were: rabbit anti-AQP4 (Millipore) 2 μg/mL and rabbit anti-Kir4.1 (Alomone Labs) 2 μg/mL. Antibodies were diluted in 0.01 M PB with 3 % normal goat serum, 1 % bovine serum albumin, 0.5 % Triton X-100, and 0.05 % sodium azide, pH 7.4. The primary antibodies were revealed by indocarbocyanine (Cy3) coupled to donkey secondary antibody (1:1,000: Jackson ImmunoResearch Laboratories, West Grove, PA) diluted in the same solution as the primary antibodies with the omission of sodium azide. Coronal sections were viewed and photographed with a Zeiss LSM 5 PASCAL microscope equipped with epifluorescence optics, using an M2 filter (BP 546/14, RKP 580, and LP 580) and 40×/1.3 Oil Plan-Neofluar objective.

## Results

**Fig. 1:**
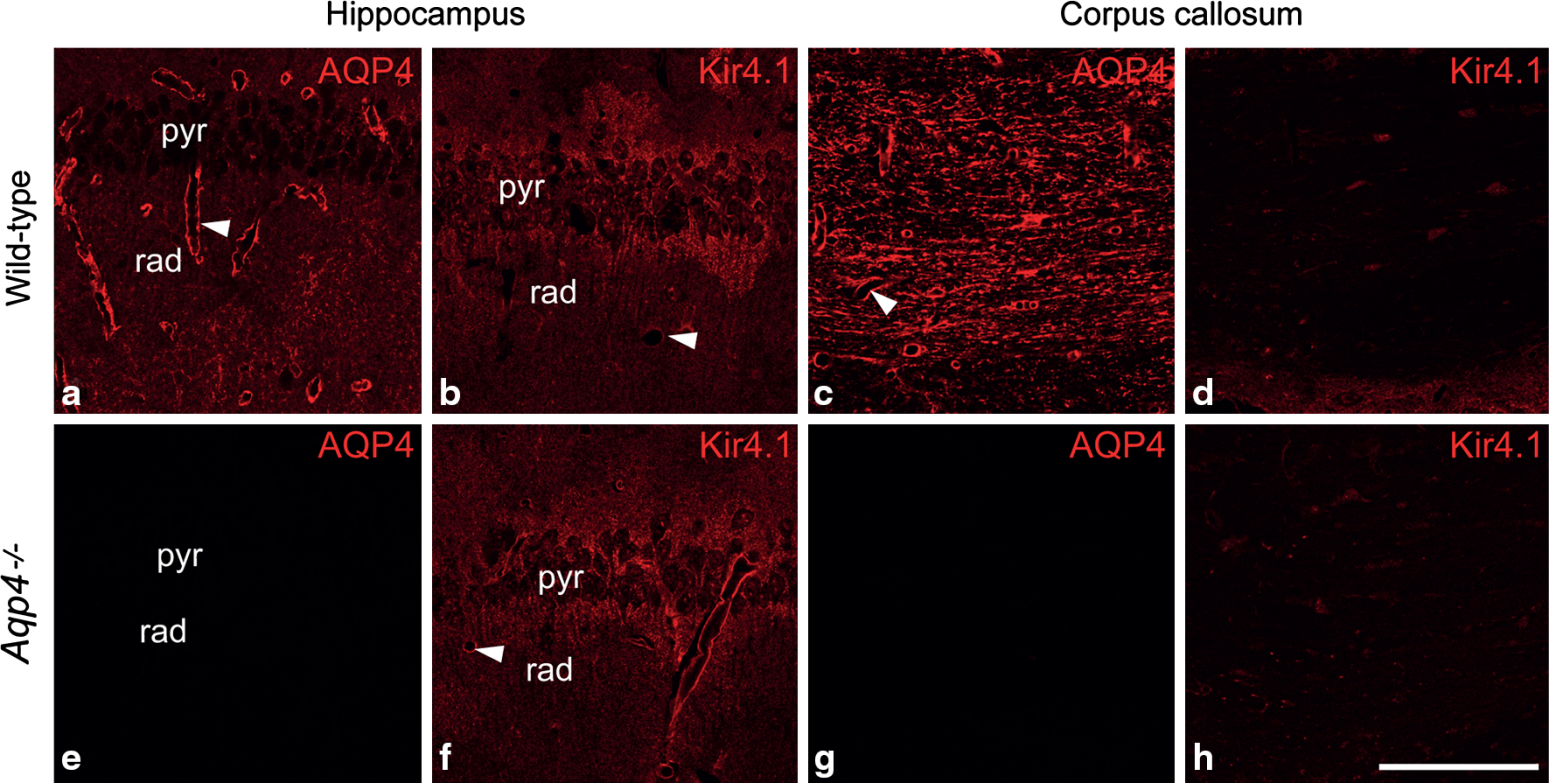
Distribution of AQP4 and Kir4.1 immunofluorescence in acute slices from hippocampus and corpus callosum. AQP4 labeling in stratum radiatum (*rad*) and stratum pyramidale (*pyr*) of the hippocampal CA1 region (**a**) and in coronal corpus callosum (**c**) from wild type mice. In both regions, a reticular staining pattern was observed, compatible with labeling of fine astrocytic processes. The intense signal around blood vessels corresponds to astrocytic endfeet (*arrowheads*). The selectivity of antibodies was confirmed by absence of AQP4 labeling in slices from *Aqp4* ^−*/*−^ mice **(e**, **g**). Kir4.1 immunofluorescence likewise outlined delicate processes resembling those of astrocytes (**b**, **d**), with less prominent perivascular signal (*arrowhead*) than observed with antibodies against AQP4. Kir4.1 immunoreactivity in hippocampus (**f**) and corpus (**h**) callosum of *Aqp4* ^−*/*−^ mice was similar to that of wild types. *Scale bar* 100 µm

AQP4 immunofluorescence of immersion fixed tissue slices revealed a reticular labeling pattern compatible with staining of astrocytic processes in both hippocampus (Fig. 1a) and corpus callosum (Fig. 1c). Intense labeling was observed around blood vessels. Absence of AQP4 labeling in *Aqp4* ^−*/*−^ mice confirmed the selectivity of antibodies (Fig. 1e, g). Kir4.1 immunoreactivity in wild type animals resembled that of AQP4, but the signal was weaker around vessels (Fig. 1b, d). Importantly, the pattern of Kir4.1 immunoreactivity in hippocampus and corpus callosum was unaffected by *Aqp4* deletion (Fig. 1f, h). The distribution of AQP4 and Kir4.1 labeling of immersion fixed slices was similar to that of perfusion fixed tissue (cf. Haj-Yasein et al. 2011).

**Fig. 2:**
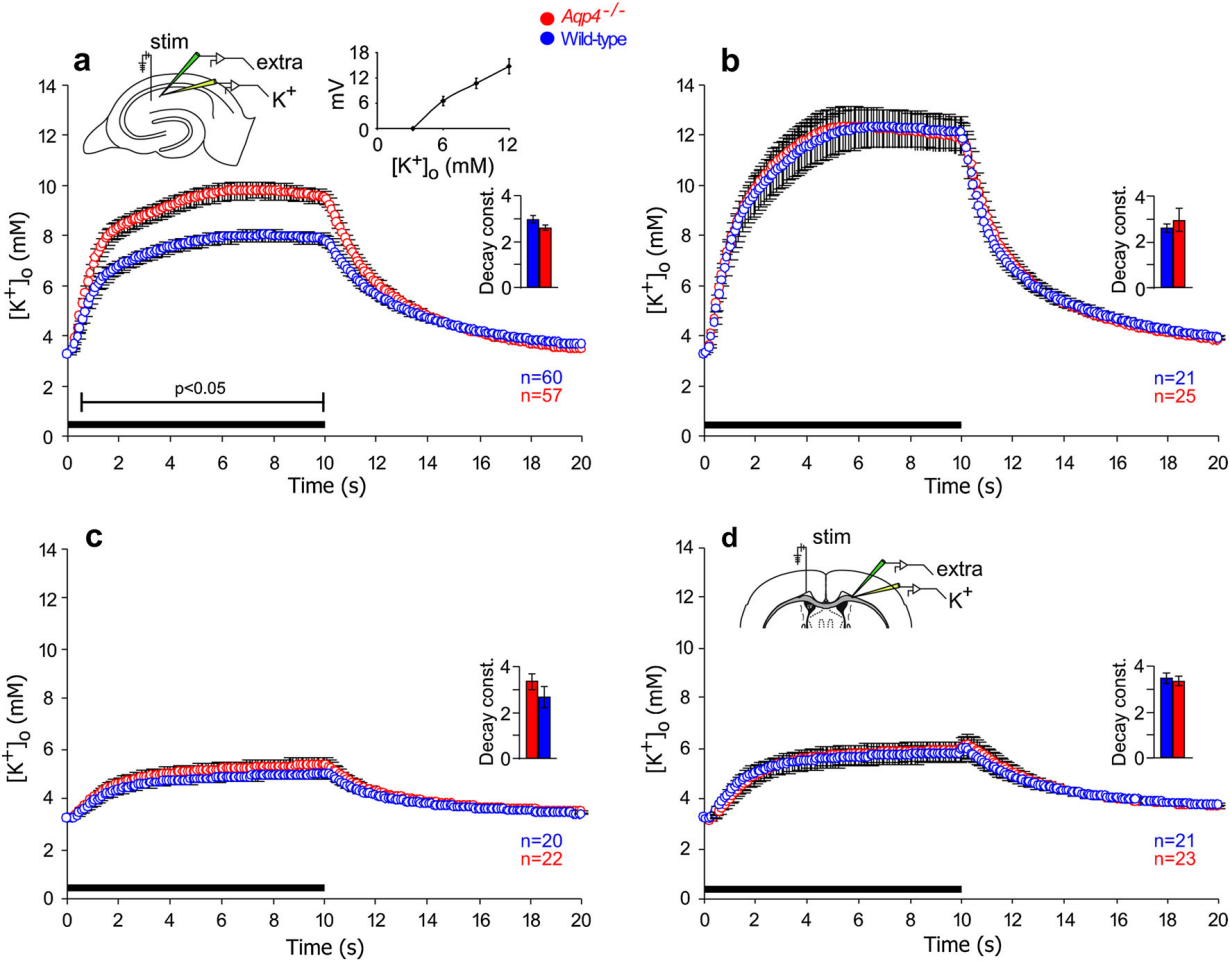
Impact of *Aqp4* deletion on extracellular K^+^ dynamics during synaptic stimulation. **a** Potassium responses during and after 10 s stimulation at 20 Hz (*black horizontal bar* along the* abscissa*) from hippocampal synaptic stratum radiatum layer CA1 of wild type (*blue circles*, *n* = 60) and *Aqp4* ^−*/*−^ mice (*red circles*, *n* = 57). *Vertical bars* indicate SEM. *Bracket* indicates period of statistical significant difference (*p* < 0.05) between genotypes. *Insets* a schematic drawing of the hippocampal formation with recording and stimulating electrodes; electrode calibration graph for the K^+^-sensitive electrodes showing the relationship between voltage and [K^+^]_o_ (*bars* indicate SD); histogram of the K^+^-decay constants, measured during the post-stimulation phase (*bars* indicate SEM). **b** As in **a**, but the recordings are from the stratum pyramidale (*n* = 21 for wild type mice, *n* = 25 for *Aqp4* ^−*/*−^ mice). **c** As in **a**, but during blockade of ionotropic glutamate receptors (50 μM AP5 and 20 μM DNQX) thus isolating the changes in [K^+^]_o_ mediated by axonal activity. The figure shows that [K^+^]_o_ during and after high-frequency stimulation was similar in the two genotypes (*n* = 20 for wild type mice, *n* = 22 for *Aqp4* ^−*/*−^ mice). **d** As in **a**, but experiments were performed on myelinated fibers of the corpus callosum. *Inset* a schematic drawing of the corpus callosum with recording and stimulating electrodes (*n* = 21 for wild type mice, *n* = 23 for *Aqp4* ^−*/*−^ mice)

The potassium-sensitive electrodes used to assess extracellular K^+^ dynamics showed voltage responses of 14.8 ± 1.8 mV (mean ± SD, *n* = 45) when [K^+^]_o_ was changed from 3.25 to 12 mM (Fig. 2a, top inset). Stimulation of Schaffer collateral/commissural fibers with parameters (20 Hz, 10 s) identical to those used to reveal effects of *Aqp4* deletion on extracellular volume dynamics (Haj-Yasein et al. 2012) elicited a robust increase in [K^+^]_o_ in the CA1 region (Fig. 2). In stratum radiatum, the peak [K^+^]_o_ during stimulation was significantly higher in *Aqp4* ^−*/*−^ animals than in wild types, whereas the [K^+^]_o_ recovery phase was similar in the two genotypes (Fig. 2a). In stratum pyramidale, neither peak [K^+^]_o_ nor post-stimulation [K^+^]_o_ recovery was affected by *Aqp4* deletion (Fig. 2b). For both genotypes, the peak [K^+^]_o_ was higher in stratum pyramidale than in stratum radiatum.

To resolve whether the effect of *Aqp4* knockout on peak [K^+^]_o_ was dependent on postsynaptic K^+^ release mediated through ionotropic glutamate receptor activation, we performed experiments in presence of the NMDA receptor antagonist AP5 (50 μM) and the AMPA receptor antagonist DNQX (20 μM). Deletion of *Aqp4* had no effect on peak [K^+^]_o_ under these conditions (Fig. 2c), where most of the released K^+^ is supposed to derive from unmyelinated axons. Similarly, we failed to detect genotype-dependent differences in [K^+^]_o_ kinetics during 20 Hz stimulation of myelinated axons in corpus callosum (Fig. 2d).

## Discussion

The present data indicate that *Aqp4* deletion leads to a significant increase in peak [K^+^]_o_ during synaptic stimulation. The peak was strongly reduced by glutamate receptor blockade, consistent with K^+^ release from postsynaptic sites. The effect of *Aqp4* deletion was restricted to the synaptic layer. Notably, the higher peak recorded at the soma layer was insensitive to *Aqp4* deletion, as was the lower peak recorded in corpus callosum.

This is the first study where the effect of *Aqp4* deletion or AQP4 mislocalization has been investigated in the synaptic termination area of a discrete anatomical pathway. Previous in vivo analyses have investigated the effect of gross cortical stimulation or cortical spreading depression (Padmawar et al. 2005; Binder et al. 2006; Thrane et al. 2013) while earlier studies of the hippocampus have focused on the soma layers (Amiry-Moghaddam et al. 2003; Strohschein et al. 2011).

Post-stimulation recovery of extracellular K^+^ did not differ between *Aqp4* ^−/−^ mice and wild types. Thus, the increased peak [K^+^]_o_ cannot reflect changes in K^+^ clearance. In agreement, the expression pattern of Kir4.1—which mediates spatial buffering (Haj-Yasein et al. 2011)—was not altered by *Aqp4* deletion. Our immunocytochemical data are complementary to the quantitative Western analysis of Zhang and Verkman (2008) who found no change in Kir4.1 following deletion of *Aqp4*. Membrane potential, barium-sensitive Kir4.1 K^+^ currents, and current–voltage relationship were likewise unchanged, as judged from recordings in freshly isolated glial cells.

The most salient explanation is that *Aqp4* deletion affects peak [K^+^]_o_ via changes in extracellular volume dynamics. This would be in line with our recent report (Haj-Yasein et al. 2012). During a stimulation paradigm identical to the present, *Aqp4* ^−/−^ animals showed a more pronounced extracellular space shrinkage than did wild type animals. Indeed, the effect on volume (Haj-Yasein et al. 2012) mimicked the effect on [K^+^]_o_ (present study), in regard to both time course and amplitude. Also, the effects of *Aqp4* deletion on volume and [K^+^]_o_ share the same layer specificity in that they occur in stratum radiatum but not in stratum pyramidale. This bolsters the idea that the effect of *Aqp4* gene deletion on peak [K^+^]_o_ is secondary to volume changes. The alternative explanation, that peak [K^+^]_o_ was increased due to enhanced excitability and K^+^ release, finds no support in previous studies (Amiry-Moghaddam et al. 2003; Haj-Yasein et al. 2012).

Analyses in slices allow precise stimulation of defined pathways and are compatible with strict control of metabolic status. Previous analyses that have demonstrated an effect of *Aqp4* deletion on extracellular K^+^ recovery were done in vivo following gross stimulations that easily could have depleted the tissue of energy substrates, thus affecting Na,K-ATPase-dependent K^+^ recovery (Padmawar et al. 2005; Binder et al. 2006; Thrane et al. 2013). Previous slice studies indicating an effect on clearance used genetic or stimulation paradigms that differed from those used here (Amiry-Moghaddam et al. 2003; Strohschein et al. 2011).

The present study shows that *Aqp4* deletion has a layer-specific effect on [K^+^]_o_ that precisely mirrors the reported effect on extracellular volume dynamics. When *Aqp4* is deleted, the stimulation induced [K^+^]_o_ will be accentuated as a direct consequence of the loss of volume homeostasis. The mechanism proposed here might explain the increase in seizure severity that is observed in animals with mislocalization or depletion of AQP4 (Amiry-Moghaddam et al. 2003; Binder et al. 2006).

## References

[CR1] Amiry-Moghaddam M, Williamson A, Palomba M, Eid T, de Lanerolle NC, Nagelhus EA, Adams ME, Froehner SC, Agre P, Ottersen OP (2003). Delayed K^+^ clearance associated with aquaporin-4 mislocalization: phenotypic defects in brains of alpha-syntrophin-null mice. Proc Natl Acad Sci USA.

[CR2] Binder DK, Yao X, Zador Z, Sick TJ, Verkman AS, Manley GT (2006). Increased seizure duration and slowed potassium kinetics in mice lacking aquaporin-4 water channels. Glia.

[CR3] Haj-Yasein NN, Jensen V, Vindedal GF, Gundersen GA, Klungland A, Ottersen OP, Hvalby O, Nagelhus EA (2011). Evidence that compromised K^+^ spatial buffering contributes to the epileptogenic effect of mutations in the human Kir4.1 gene (KCNJ10). Glia.

[CR4] Haj-Yasein NN, Jensen V, Ostby I, Omholt SW, Voipio J, Kaila K, Ottersen OP, Hvalby O, Nagelhus EA (2012). Aquaporin-4 regulates extracellular space volume dynamics during high-frequency synaptic stimulation: a gene deletion study in mouse hippocampus. Glia.

[CR5] Illarionova NB, Gunnarson E, Li Y, Brismar H, Bondar A, Zelenin S, Aperia A (2010). Functional and molecular interactions between aquaporins and Na, K-ATPase. Neuroscience.

[CR6] Kofuji P, Newman EA (2004). Potassium buffering in the central nervous system. Neuroscience.

[CR7] Nagelhus EA, Ottersen OP (2013). Physiological roles of aquaporin-4 in brain. Physiol Rev.

[CR8] Nagelhus EA, Horio Y, Inanobe A, Fujita A, Haug FM, Nielsen S, Kurachi Y, Ottersen OP (1999). Immunogold evidence suggests that coupling of K^+^ siphoning and water transport in rat retinal Muller cells is mediated by a coenrichment of Kir4.1 and AQP4 in specific membrane domains. Glia.

[CR9] Nielsen S, Nagelhus EA, Amiry-Moghaddam M, Bourque C, Agre P, Ottersen OP (1997). Specialized membrane domains for water transport in glial cells: high-resolution immunogold cytochemistry of aquaporin-4 in rat brain. J Neurosci.

[CR10] Padmawar P, Yao X, Bloch O, Manley GT, Verkman AS (2005). K^+^ waves in brain cortex visualized using a long-wavelength K^+^ -sensing fluorescent indicator. Nat Methods.

[CR11] Strohschein S, Huttmann K, Gabriel S, Binder DK, Heinemann U, Steinhauser C (2011). Impact of aquaporin-4 channels on K(+) buffering and gap junction coupling in the hippocampus. Glia.

[CR12] Thrane AS, Rappold PM, Fujita T, Torres A, Bekar LK, Takano T, Peng W, Wang F, Thrane VR, Enger R, Haj-Yasein NN, Skare O, Holen T, Klungland A, Ottersen OP, Nedergaard M, Nagelhus EA (2011). Critical role of aquaporin-4 (AQP4) in astrocytic Ca^2+^ signaling events elicited by cerebral edema. Proc Natl Acad Sci USA.

[CR13] Thrane AS, Takano T, Thrane VR, Wang F, Peng W, Ottersen OP, Nedergaard M, Nagelhus EA (2013) In vivo NADH fluorescence imaging indicates effect of aquaporin-4 deletion on oxygen microdistribution in cortical spreading depression. J Cereb Blood Flow Metab 10.1038/jcbfm.2013.63 PMC3705443 23611872

[CR14] Zhang H, Verkman AS (2008). Aquaporin-4 independent Kir4.1 K^+^ channel function in brain glial cells. Mol Cell Neurosci.

